# Multimodal image translation via deep learning inference model trained in video domain

**DOI:** 10.1186/s12880-022-00854-x

**Published:** 2022-07-14

**Authors:** Jiawei Fan, Zhiqiang Liu, Dong Yang, Jian Qiao, Jun Zhao, Jiazhou Wang, Weigang Hu

**Affiliations:** 1 Department of Radiation Oncology, Fudan University Shanghai Cancer Center, Shanghai, 200032 People’s Republic of China; 2 Department of Oncology, Shanghai Medical College Fudan University, Shanghai, 200032 People’s Republic of China; 3 Shanghai Key Laboratory of Radiation Oncology, Shanghai, 200032 People’s Republic of China; 4 National Cancer Center/National Clinical Research Center for Cancer/Cancer Hospital, Chinese Academy of Medical Sciences and Peking Union Medical College, Beijing, China

**Keywords:** Video domain, Deep learning, Medical image translation, GAN

## Abstract

**Background:**

Current medical image translation is implemented in the image domain. Considering the medical image acquisition is essentially a temporally continuous process, we attempt to develop a novel image translation framework via deep learning trained in video domain for generating synthesized computed tomography (CT) images from cone-beam computed tomography (CBCT) images.

**Methods:**

For a proof-of-concept demonstration, CBCT and CT images from 100 patients were collected to demonstrate the feasibility and reliability of the proposed framework. The CBCT and CT images were further registered as paired samples and used as the input data for the supervised model training. A vid2vid framework based on the conditional GAN network, with carefully-designed generators, discriminators and a new spatio-temporal learning objective, was applied to realize the CBCT–CT image translation in the video domain. Four evaluation metrics, including mean absolute error (MAE), peak signal-to-noise ratio (PSNR), normalized cross-correlation (NCC), and structural similarity (SSIM), were calculated on all the real and synthetic CT images from 10 new testing patients to illustrate the model performance.

**Results:**

The average values for four evaluation metrics, including MAE, PSNR, NCC, and SSIM, are 23.27 ± 5.53, 32.67 ± 1.98, 0.99 ± 0.0059, and 0.97 ± 0.028, respectively. Most of the pixel-wise hounsfield units value differences between real and synthetic CT images are within 50. The synthetic CT images have great agreement with the real CT images and the image quality is improved with lower noise and artifacts compared with CBCT images.

**Conclusions:**

We developed a deep-learning-based approach to perform the medical image translation problem in the video domain. Although the feasibility and reliability of the proposed framework were demonstrated by CBCT–CT image translation, it can be easily extended to other types of medical images. The current results illustrate that it is a very promising method that may pave a new path for medical image translation research.

## Introduction

In the field of medical imaging, a wide range of methods are used to obtain spatially resolved information about patient anatomy. This includes plain radiography, computed tomography (CT), magnetic resonance imaging (MRI), positron emission tomography (PET) and cone-beam computed tomography (CBCT) used for enhanced image-guided radiation therapy. Due to the different underlying physical principles, these image data are of different dimensionality and varying contrasts. Although this variety offers various diagnostic options, the generation of all these image data for one patient may not be feasible in specific situations. For example, CBCT, alternative to CT, is routinely used in clinic to provide accurate volumetric imaging of the treatment position for patient setup during online adaptive radiotherapy. However, the CT number in CBCT is not accurate enough, which is due to cupping and scattering artifacts caused by the large illumination field, for soft tissue-based patient setup and further quantitative applications such as dose calculation and adaptive treatment planning. Therefore, a framework which is capable of translating between multiple modalities would shorten the medical procedure by removing additional unnecessary scans and provide additional medical information. It poses several challenges, but enhances medical efficiency and is proved to be beneficial for both medical professionals and patients [1–3]. Therefore, medical image translation with numerous potential applications is considered as a new frontier in the field of medical image analysis.

Recently, with the development of deep learning algorithms, especially the convolutional neural network (CNN), medical image analysis has made significant progress in a range of applications such as lesion detection and classification [4, 5], image registration and enhancement [6, 7], organs segmentation [8, 9], and dose calculation in radiotherapy [10–12]. This also led to the development of several deep neural networks based approaches for the translation of medical images [13–18]. The most prominent of these deep neural networks are Generative Adversarial Networks (GANs) [19]. The main underlying principle of GANs is that of the competition between two co-existing networks, the generator and the discriminator, which are trained simultaneously with opposing goals. The generator network generates synthetic data samples, while the discriminator network acts as a binary classifier attempting to distinguish between real training samples and generated synthetic samples. More specifically, the generator is intended to maximize the probability of fooling the discriminator into considering the synthetic samples are realistic, while the discriminator is trained to maximize the probability of correctly classifying real and synthetic samples, thus minimize the differences between them [19]. Pix2pix GAN framework, in which the generator is a U-Net based architecture and the discriminator is represented by a convolutional PatchGAN classifier, is a general solution to supervised image-to-image translation problems [20]. It was utilized to translate MR to CT images [21], denoise low dose CT images by translating it into a high dose counterpart [22], and perform multi-leaf collimator shape images generation for radiotherapy [11]. Cycle-GAN framework [23], which involves the simultaneous training of two generator models and two discriminator models with the cycle consistency loss, is another similar but unsupervised approach. It was first applied to translate MR to CT images on head and neck sites [24], then applied on pelvic sites with more training data and incorporating gradient consistency loss [25]. It was also trained to learn translation functions from a source domain CBCT to a target domain CT with high accuracy and efficiency in radiotherapy related studies [17].

The aforementioned studies take assumption that the patient’s whole image volume, regardless of MR, CT or CBCT, is consisting of multiple isolated slices, thus the translation is fulfilled in the image domain. Noted that patient’s image scan is a temporally continuous process, some inherent spatio-temporal correlations and extra context information present between individual slices but cannot be considered in the image domain translation. Taking another perspective, if we treat one slice as one frame, then the patient whole image volume can be considered as a video, can we fulfill the translation in the video domain and obtain better results by accurately modeling the temporal dynamics and other context information? Under the guidance of this concept, we propose a new medical image translation method based on a video-to-video synthesis approach. This method considers the patient whole image volume as a complete and independent unit, takes the spatio-temporal correlations among the individual slices into account, and performs the medical image translation from a new perspective. To our knowledge, this is the first attempt of applying the video concept in medical image translation research. We compared the performance of the proposed framework with our previously published Cycle-GAN model and the results show that the new method outperforms the existing model.

## Methods

### Data collection and preprocessing

For a proof-of-concept demonstration, we collected rectum cancer patients’ CT and CBCT images as our datasets to evaluate the proposed method. A new CT-linac uRT-linac 506c designed by United Imaging Healthcare Co. Ltd, which integrated a diagnostic-quality 16-slices helical CT and a C-arm linac together, was used for data acquisition [17]. The helical CT can be used for simulation, and the electronic portal imaging detector (EPID) system was used for 3D MV CBCT acquisition. These images were acquired almost in the same position and anatomy, and further registered between each other to prevent producing randomized hallucinate anatomy that is commonly seen in the unpaired image translation [26]. Noted that there are still minor mismatches after registration between the images, but it is proved that the deep neural network is relatively robust to these small perturbations.

Before images were fed into the model, all the CT and CBCT images were resampled to the size of 512 × 512 with the resolution of 0.88 × 0.88 mm^2^. The thickness of these image is 3 mm and only the image slices that presented in both CT and CBCT volumes were retained in the final dataset. The discarded slices are all at the marginal area and should not contain any important diagnostic information. To speed up the training convergence, we scaled the CT and CBCT image pixel values to the range of (-1, 1) according to the formula ${P}_{new}=2\times \frac{{P}_{original}+1000}{3048}-1$, where ${P}_{original}$indicates the original image pixel values locating in the range of (-1000, 2048). We collected CT and CBCT images from 100 patients for model training [17], and the images from the other 10 new patients were used for model evaluation.

### Image translation based on video-to-video synthesis

We converted the image translation to a video-to-video synthesis problem by considering the patient whole image volume as a video consisting of several frames (image slices). The video-to-video synthesis is essentially a distribution matching problem, the goal of which is to train a model such that the conditional distribution of the synthesized videos given input videos resembles that of real videos. To this end, the vid2vid framework [27] was applied as a basic neural network to fulfill this task. It is a conditional GAN-based model, with carefully-designed generators, discriminators and a new spatio-temporal learning objective, which is capable of synthesizing high accurate and temporally coherent videos.

**Fig. 1:**
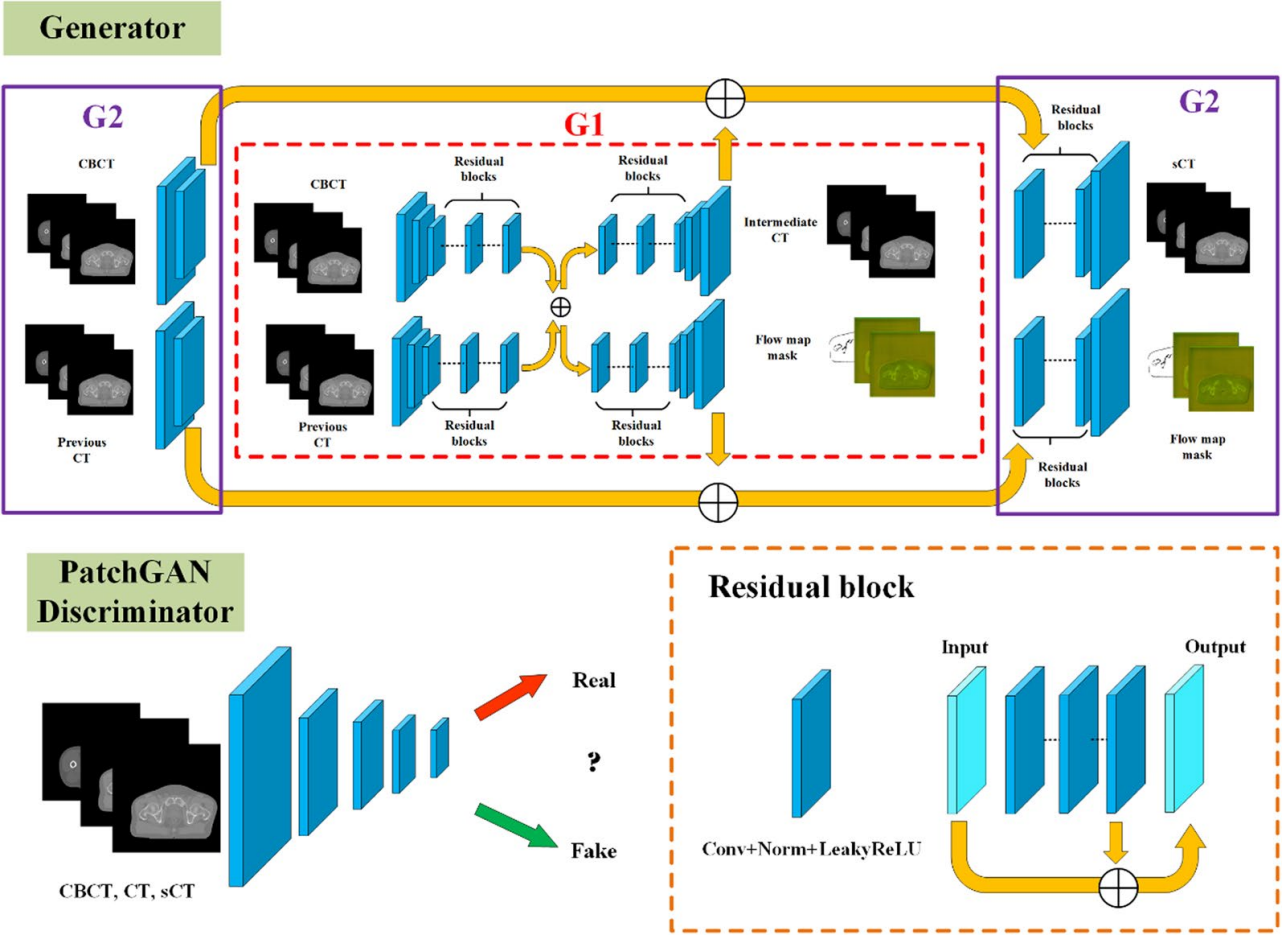
The architecture of the proposed video-to-video synthesis framework

We assume the video frames can be generated sequentially, and the generation of the current frame only depends on three factors: current source frame, past L source frames and past L generated frames, L is set to 2 after careful study. This sequential generator adopts a coarse-to-fine architecture in which the number of spatial scales is set to 2. For the lower resolution, here is 256 × 256, the network G1 shown in Fig. 1 takes two source frames and previously generated frames as inputs. They first undergo two separate downsampling residual blocks, then the intermediate high-level features are added and fed into each unsampling residual blocks to output the lower resolution generated frame as well as the flow map and occlusion mask. The flow map displays the estimated optical flow between consecutive frames and the occlusion mask is used to add new texture details by gradually blending the warped pixels and the newly synthesized pixels [27]. Next, another higher resolution network G2 with similar architecture is built on the top of the network G1. It first downsamples the inputs and feeds them into G1, then the extracted features from the last layer of G1 are added to the intermediate layers of G2, these summed features are finally fed into upsampling residual blocks to output the new generated frame [27]. The hyperparameters are determined by checking the model performance, nine residual blocks consisting of convolutional, normalization and activation layers are used as backbones of aforementioned networks G1 and G2. The kernel size and largest filter size is set to 3 and 512 respectively to fit the GPU memory.

Two discriminators, the image discriminator D_I_ and the video discriminator D_V_, are implemented in the proposed framework. The image discriminator D_I_ which adopts the multi-scale PatchGAN architecture ensures that each output frame resembles a real image given the same source image. The temporally multi-scale video discriminator D_V_ downsamples the frame rates (skipping every three intermediate frames) of the real/generated videos for up to three scales and helps ensure that consecutive output frames resemble the temporal dynamics of a real video given the same optical flow [27]. The two discriminators are built upon the same convolutional blocks shown in Fig. 1. The kernel size and largest filter size is set to 4 and 256 respectively to fit the GPU memory.

Three losses are defined, namely the image-conditional GAN loss ${\mathcal{L}}_{I}$, the video GAN loss ${\mathcal{L}}_{V}$, and the optical flow estimation loss ${\mathcal{L}}_{F}$ [27]. To make the generated frames indistinguishable from the real frames, we adopted ${\mathcal{L}}_{I}$ loss defined by image discriminator D_I_:

1 $$  {\mathcal{L}}_{I}  = {~\mathbb{E}}_{{x_{t} ,s_{t} }} \left[ {\log D_{I} \left( {x_{t} ,s_{t} } \right)} \right] + ~{\mathbb{E}}_{{\tilde{x}_{t} ,~s_{t} }} \left[ {\log \left( {1 - D_{I} \left( {\tilde{x}_{t} ,~s_{t} } \right)} \right)} \right] $$

where ${s}_{t},{ x}_{t}, {\tilde{x}}_{t}$ denote source video frames, real video frames and generated video frames, respectively. Similarly, to make the K consecutive generated frames resemble the real videos, we defined ${\mathcal{L}}_{V}$ loss:

2 $$   {\mathcal{L}}_{{V~}}  = ~{\mathbb{E}}_{{w_{{t - K}}^{{t - 2}} ,x_{{t - K,}}^{{t - 1}} s_{{t - K}}^{{t - 1}} }} ~\left[ {\log D_{V} \left( {x_{{t - K}}^{{t - 1}} ,w_{{t - K}}^{{t - 2}} } \right)} \right] + ~{\mathbb{E}}_{{w_{{t - K}}^{{t - 2}} ,\tilde{x}_{{t - K,}}^{{t - 1}} s_{{t - K}}^{{t - 1}} }} \left[ {\log \left( {1 - D_{V} \left( {\tilde{x}_{{t - K}}^{{t - 1}} ,w_{{t - K}}^{{t - 2}} } \right)} \right)} \right]   $$

where ${s}_{t-K}^{t-1}, { w}_{t-K}^{t-2},{ x}_{t-K,}^{t-1}{ \tilde{x}}_{t-K}^{t-1}$ denote K consecutive source video frames, K-1 optical flow, K real video frames and K generated video frames, respectively. During model training, K doubles sequentially from 4 until the total number of frames. Finally, the flow estimation loss ${\mathcal{L}}_{F}$ was given by two terms, the first term denotes the difference between the real and the estimated optical flow, the second term is warping difference between the next real frame and next optical flow warped frame:

3 $${\mathcal{L}}_{F}= \frac{1}{T-1}\sum _{t=1}^{T-1}\left({\|{\tilde{w}}_{t}- {w}_{t}\|}_{1}+{\|{\tilde{w}}_{t}\left({x}_{t}\right)- {x}_{t+1}\|}_{1}\right)$$

where $\left\| \cdot \right\|_{1}$ is L1-norm, and ${\tilde{w}}_{t}, {w}_{t}, {\tilde{w}}_{t}\left({x}_{t}\right), {x}_{t+1}$ denote the estimated optical flow, real optical flow, optical flow warped next frame based on the previous frame ${x}_{t}$, and the real next frame, respectively. Hence, the model training process is essentially solving:

4 $$ \mathop {\min }\limits_{G} \left( {\mathop {\max }\limits_{{D_{I} }} \;{\mathcal{L}}_{I} \left( {G,D_{I} } \right) + ~\mathop {\max \;}\limits_{{D_{V} }} {\mathcal{L}}_{V} \left( {G,~D_{V} } \right)} \right) + ~\lambda _{F} {\mathcal{L}}_{F} \left( G \right) $$

where G denotes the sequential generator, ${\lambda }_{F}$ is a hyperparameter that balances the contributions from different losses and is set to 10 after careful study.

## Implementation and evaluation

To optimize our networks, the Adam solver was implemented and the learning rate was set to 0.0001. Early stopping was adopted by evaluating the model performance and it takes about one week for model training. However, only about 10 s are sufficient to generate the patient whole synthetic CT volume which makes it possible for clinical application. The framework was implemented by the open source deep learning library PyTorch [28] and the model was trained and tested on two Nvidia RTX 2080Ti GPUs with 11 GB VRAM.

For the synthetic image quality evaluation, we used mean absolute error (MAE), peak signal-to-noise ratio (PSNR), normalized cross-correlation (NCC), and structural similarity (SSIM) as evaluation metrics. The definitions are presented as follows.

5 $$MAE=\frac{1}{{n}_{i}{n}_{j}}\sum _{x,y}^{{n}_{i}{,n}_{j}}|{I}_{1}\left(x,y\right)-{I}_{2}(x,y\left)\right|$$

6 $$PSNR=10\times {\text{log}}_{10}\left(\frac{{MAX}^{2}}{\sum _{x,y}^{{n}_{i,}{n}_{j}}{|{I}_{1}\left(x,y\right)-{I}_{2}(x,y\left)\right|}^{2}/{n}_{i}{n}_{j}}\right)$$

7 $$   NCC = ~\frac{1}{{n_{i} n_{j} }}\mathop \sum \limits_{{x,y}}^{{n_{i} ,n_{j} }} \frac{{\left( {I_{1} \left( {x,y} \right) - \mu _{{I_{1} }} } \right)\left( {I_{2} \left( {x,y} \right) - \mu _{{I_{2} }} } \right)}}{{\sigma _{{I_{1} }} \sigma _{{I_{2} }} }} $$

8 $$SSIM= \frac{\left(2{\mu }_{{I}_{1}}{\mu }_{{I}_{2}}+{c}_{1}\right)\left(2{\sigma }_{{I}_{1},{I}_{2}}+{c}_{2}\right)}{\left({\mu }_{{I}_{1}}^{2}+{\mu }_{{I}_{2}}^{2}+{c}_{1}\right)\left({\sigma }_{{I}_{1}}^{2}+{\sigma }_{{I}_{2}}^{2}+{c}_{2}\right)}$$

where ${I}_{1}$and ${I}_{2}$ denote two different images, including ground truth and synthetic images. $I\left(x,y\right)$ means the hounsfield units (HU) value of the pixel $(x,y)$ in image $I$. ${n}_{i}{n}_{j}$is the total number of pixels in image $I$. $MAX$ is the maximum HU value in the selected image. µ and σ represent the mean and the standard deviation of the HU value in an image. ${c}_{1}$ and ${c}_{2}$ are predefined constants. The evaluation was performed on all the images from 10 testing patients and statistical analysis was also performed to demonstrate their consistency.

## Results

**Fig. 2:**
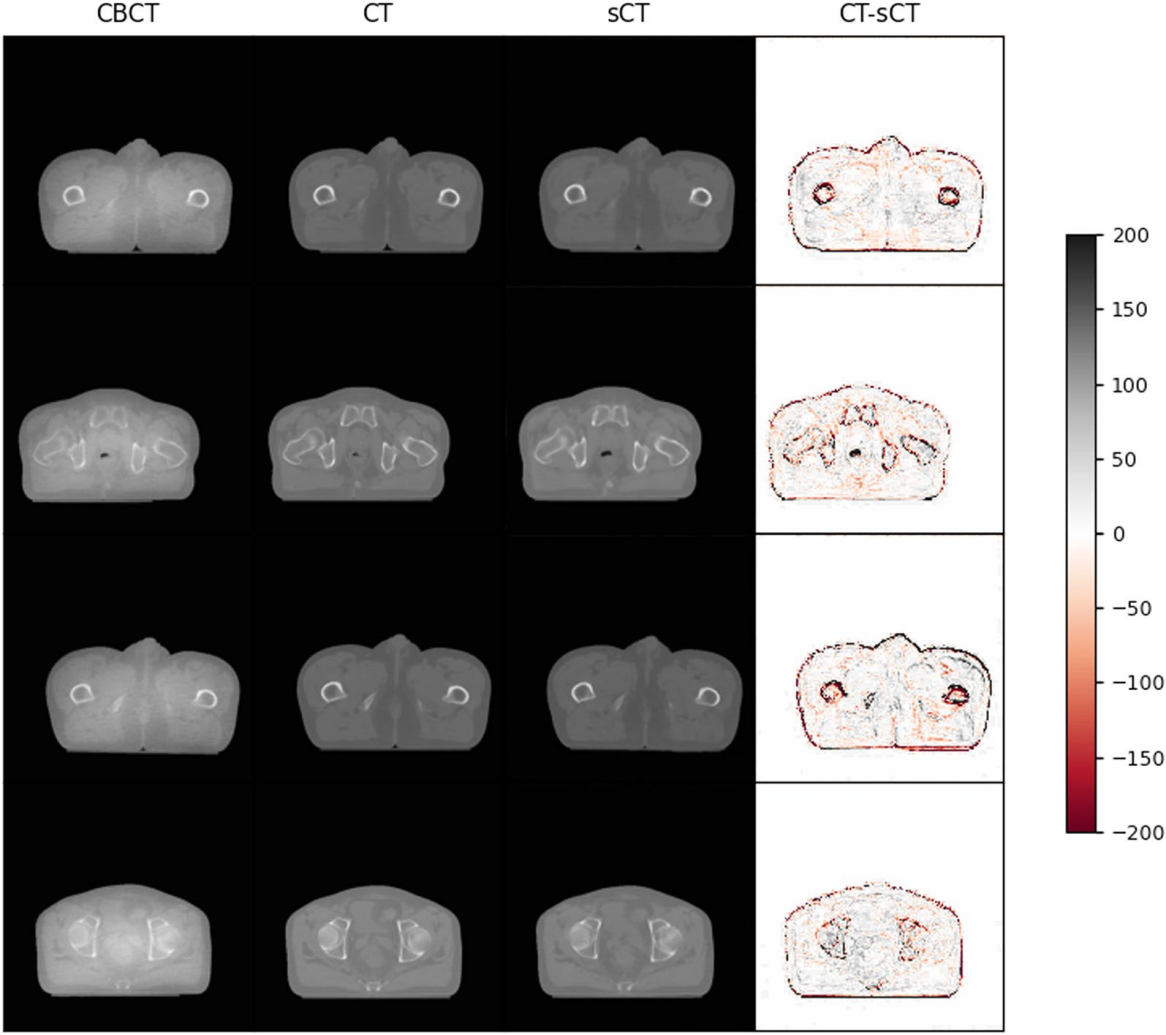
CBCT, CT and synthetic CT (sCT) images comparison from four different testing patients with different anatomical structures. The first column is CBCT images, the second column is real CT images, the third column is synthetic CT images and the last column is pixel-wise differences between real and synthetic CT images

**Fig. 3:**
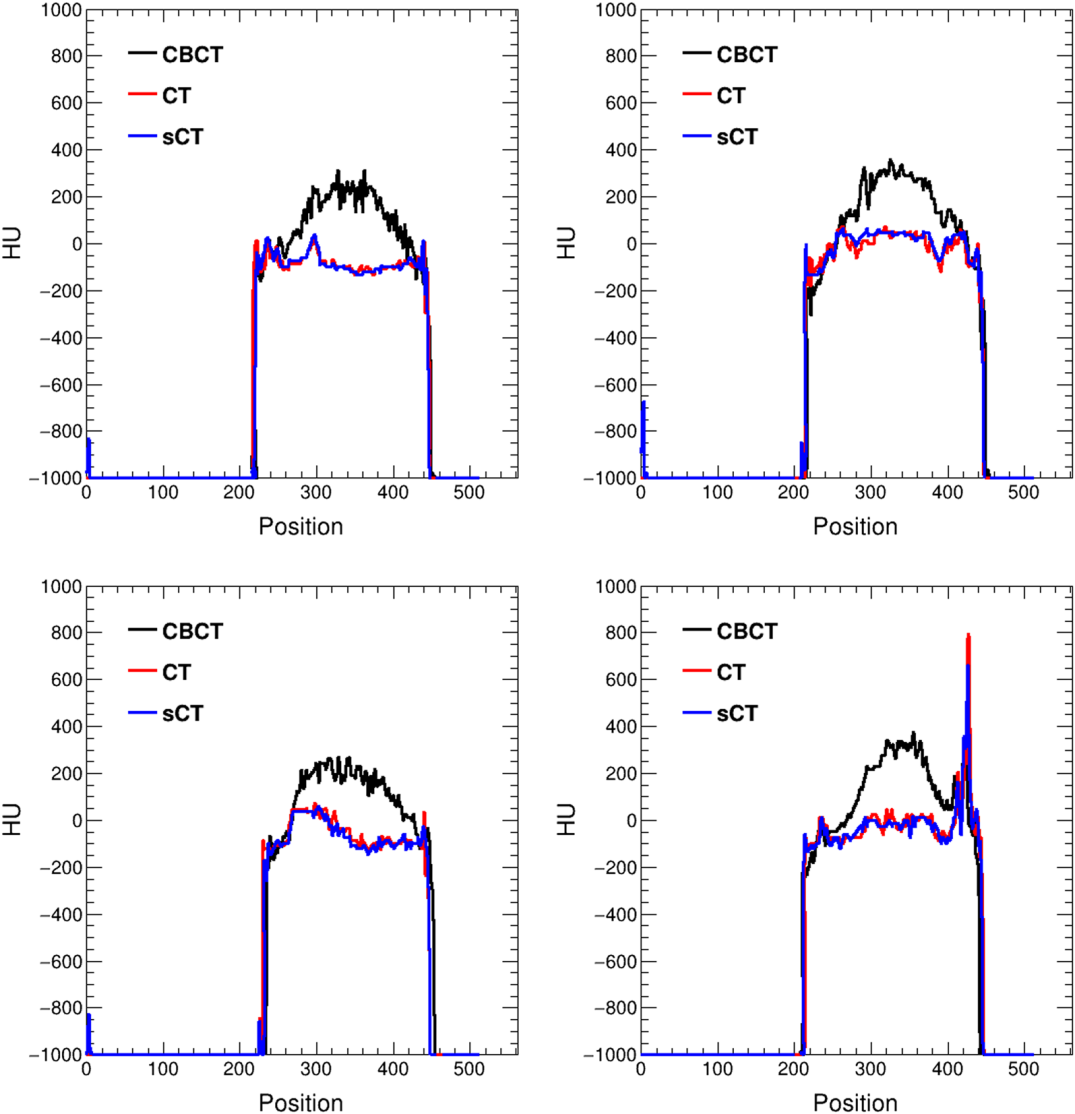
HU line profiles, through the center of the images in Fig. 2, comparison between real CT (red line), synthetic CT (blue line) and CBCT (black line) images

To evaluate the performance of the proposed image translation neural network, we show four synthetic CT images obtained from four different testing patients with different anatomical structures in Fig. 2. The first column is CBCT images, the second column is real CT images, the third column is synthetic CT images and the last column is pixel-wise differences between real and synthetic CT images. Less artifacts are found in all the synthetic CT images than in the original CBCT images and the synthetic CT images quality is very close to the real CT images. Figure 3 illustrates the good agreement of the HU line profiles, through the center of the images in Fig. 2, between real (red line) and synthetic (blue line) CT images, the HU line profiles for CBCT (black line) images are also presented to demonstrate the significant discrepancy from the others.

**Fig. 4:**
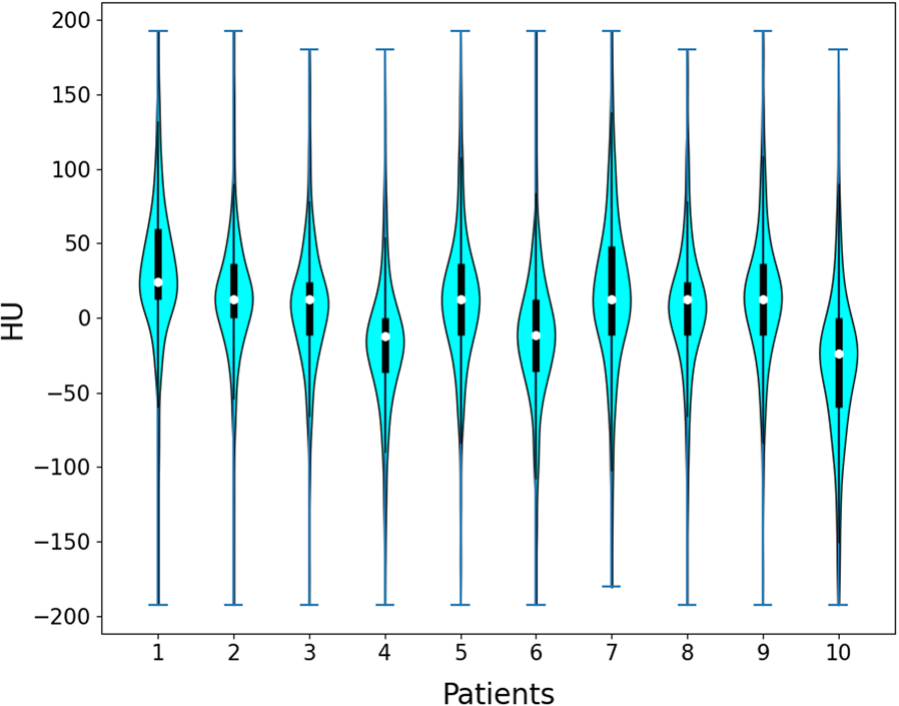
Violin plot illustrating the distribution of all the pixel-wise differences in all the images from each testing patient

For the 10 testing patients, a violin plot Fig. 4 was drawn to illustrate the distribution of all the pixel-wise differences in all the images from each testing patient. It can be seen that most of the pixel-wise HU value differences are within 50. As listed in Table 1, the aforementioned four evaluation metrics were calculated for CBCT, real and synthetic CT images. Their values for CBCT versus CT and synthetic CT versus CT are improved from 46.68 ± 9.25, 28.05 ± 1.21, 0.97 ± 0.0084, 0.92 ± 0.014 to 23.27 ± 5.53, 32.67 ± 1.98, 0.99 ± 0.0059, 0.97 ± 0.028, respectively. The previously published Cycle-GAN model [17] testing results are also presented in the last column for the detailed comparison. Significantly better image quality evaluation values are presented in synthetic CT images compared with CBCT images. In addition, we found that the image quality of synthetic CT generated by the proposed video-to-video synthesis framework was significantly improved compared with our previously published paper which utilizes Cycle-GAN to perform image translation [17]. It indicates that performing image translation in the video domain by accurately modeling the inherent spatio-temporal correlations presenting between individual slices is promising and will be a new perspective to fulfill image translation task.

**Table 1:** Four evaluation metrics values calculated from CBCT and CT, CT and synthetic CT (sCT)

	CBCT vs. CT	sCT vs. CT	sCT vs. CT (Cycle-GAN)
MAE	46.68 ± 9.25	23.27 ± 5.53	26.46 ± 6.13
PSNR	28.05 ± 1.21	32.67 ± 1.98	31.81 ± 1.82
NCC	0.97 ± 0.0084	0.99 ± 0.0059	0.98 ± 0.009
SSIM	0.92 ± 0.014	0.97 ± 0.028	0.94 ± 0.037

## Discussion

We formulated the medical image translation problem in the video domain where a conditional GANs based video-to-video synthesis framework, consisting of carefully-designed generators and discriminators as well as a spatio-temporal adversarial objective, was employed. Conditional GANs were chosen as a backbone of the framework because of its outstanding performance for image generation, and a spatio-temporal adversarial objective was implemented to guarantee the generation of the temporally consistent video. The CBCT to CT translation task was adopted as an illustration to demonstrate the feasibility and reliability of the proposed framework. However, the proposed framework can be easily extended to other medical image translation task by changing the input of the neural network. To our knowledge, this is the first work that fulfills the medical image translation task in the video domain and extensive testing demonstrates that our results are better than the results utilizing the Cycle-GAN framework in image domain.

The proposed framework uses paired training data that allows the algorithm to focus on reducing image artifacts and enhancing soft tissue contrast, rather than focusing on the large geometric mismatches. It also speeds up the training model because of the reduced relative differences from the beginning. In practice, the video frames are generated sequentially by taking the optical flow between consecutive frames into account. Different from the conventional image domain GAN-based networks, two discriminators are adopted in the framework. The image discriminator D_I_ ensures that each output frame resembles a real image, and the video discriminator D_V_ which is unique in the proposed framework ensures that consecutive output frames resemble a real video and helps guarantee both short-term and long-term consistency. The proposed framework integrates the patient individual images as a video, considers the spatio-temporal correlations among them, and proves to outperform than the existing method defined in the image domain.

Our results show that the synthetic CT images have great agreement with the real CT images and the image quality is improved with lower noise and artifacts compared with CBCT images. The pixel-wise differences are slightly larger in some regions shown in Fig. 2, this is because the training data locating in the abdominal region is easily affected by respiratory motion, organ movement and organ filling status. However, most of the pixel-wise HU value differences are within 50, seen from Fig. 4, which is essentially small enough for clinical practice. Compared with the Fig. 4 in our previously published results [17], the average absolute pixel-wise difference significantly reduced from about 200 to 50. The HU line profiles also present better agreements between CT and synthetic CT compared with previously published results [17]. Meanwhile, the aforementioned four evaluation metrics are better compared with the results obtained from the previously trained Cycle-GAN model, seen from Table 1. The essential difference between them is the network training domain, the proposed video training domain integrating extra spatio-temporal correlations among images was proved to be capable for more accurate and stable translation results.

In this study, we implemented a CBCT to CT image translation task by training a framework defined in the video domain. For a proof-of-concept demonstration, we only studied the model performance on CBCT and CT data, additional medical images are required to further investigate the model generality and universality. We believe it could be easily extended to other types of medical image translation tasks including MRI-CT, PET-CT translation and so on. This will be one of our future investigations. Meanwhile, the current framework is limited to paired training samples, another crucial study is to improve the framework and allow the unpaired image translation. Additionally, we plan on expanding the framework with other backbone networks, optimizing the architecture by performing some ablation studies to verify the effectiveness of each network component, and improving the training efficiency for more complex data. Furthermore, we plan to add more comparison studies to include performance on clinical applications, such as dose calculation and diagnostic application.

## Conclusions

In this work, we pioneered to propose a new perspective for medical image translation in the video domain. Its feasibility and reliability were demonstrated by CBCT-CT image translation and can be easily extended to other types of medical images. Future work will be focused on evaluating the method on different datasets and further improving accuracy. It is a very promising method that may pave a new path for medical image translation research.

## Data Availability

The datasets analysed during the current study are not publicly available due to the hospital policy but are available from the corresponding author on reasonable request.

## Abbreviations

CBCT: Cone-beam computed tomography
CT: Computed tomography
MAE: Mean absolute error
PSNR: Peak signal-to-noise ratio
NCC: Normalized cross-correlation
SSIM: Structural similarity
MRI: Magnetic resonance imaging
PET: Positron emission tomography
HU: Hounsfield units
CNN: Convolutional neural network
GANs: Generative adversarial networks
EPID: Electronic portal imaging detector
